# Temporal association between adjunctive repetitive transcranial magnetic stimulation and seizure reduction in a pediatric patient with DNM1L-associated EMPF1: a case report

**DOI:** 10.3389/fped.2026.1933161

**Published:** 2026-09-30

**Authors:** Xianghui Chen, Pinglian Sun, Xiaohong Ren, Gege Liu, Xiaoyuan Bai, Feng Guo, Rui Guo, Shirun Wu, Huifang Wang

**Affiliations:** 1 Department of Critical Care Medicine, Lvliang First People's Hospital, Lvliang Hospital of Shanxi Medical University, Lvliang, China; 2 Department of Electrophysiology, Lvliang First People's Hospital, Lvliang Hospital of Shanxi Medical University, Lvliang, China; 3 Department of Neurology, Lvliang First People's Hospital, Lvliang Hospital of Shanxi Medical University, Lvliang, China; 4 Department of Psychiatry, Lvliang First People's Hospital, Lvliang Hospital of Shanxi Medical University, Lvliang, China; 5 Department of Pediatrics, First Hospital of Shanxi Medical University, Shanxi, China; 6 Department of Neurology, First Hospital of Shanxi Medical University, Shanxi, China

**Keywords:** DNM1L mutation, EMPF1, mitochondrial disease, myoclonic seizures, refractory epilepsy, repetitive transcranial magnetic stimulation

## Abstract

**Background:**

Encephalopathy, lethal, due to defective mitochondrial and peroxisomal fission 1 (EMPF1) is a rare, severe mitochondrial disorder characterized by acute-onset refractory seizures that rapidly progress to status epilepticus and are associated with a poor prognosis. Conventional therapies, including antiepileptic drugs and the ketogenic diet, exhibit limited efficacy. Although repetitive transcranial magnetic stimulation (rTMS) has shown promise in other forms of drug-resistant epilepsy, its potential role in managing EMPF1 remains unexplored.

**Case presentation:**

A 7-year-old boy, previously healthy with normal development, presented with right-hand clonic movements and impaired consciousness, which progressed to status epilepticus. Brain MRI revealed diffuse cytotoxic edema involving the bilateral frontal, parietal, and temporal cortices, basal ganglia, and thalami. EEG recording showed generalized periodic spike-wave discharges with background slowing. Genetic testing identified a novel *de novo* DNM1L mutation (c.1949T > C, p.Leu650Pro), which was classified as likely pathogenic according to ACMG/AMP guidelines and provided strong support for the diagnosis of EMPF1. In addition to a regimen of antiepileptic drugs and a metabolic “cocktail,” 1-Hz rTMS (90% of the resting motor threshold, 40 trains daily) was applied over the left primary motor cortex on Day 16. Following initiation of this combined therapy, seizure frequency was temporally associated with a marked reduction, decreasing from ∼20 to 5–6 episodes/day after one day, 2–3 by Day 27 (12 days after rTMS initiation), and 0–1 by Day 36 (21 days after rTMS initiation). Despite this reduction in clinical seizure burden, the patient developed progressive pneumonia leading to sepsis and acute heart failure, and died on Day 65 (hospital day 65).

**Conclusions:**

In this case, adjunctive rTMS applied alongside standard antiepileptic and metabolic “cocktail” therapy was temporally associated with a pronounced reduction in seizure frequency in a patient with DNM1L-associated EMPF1. This temporal association is hypothesis-generating and does not permit attribution of the observed seizure reduction to rTMS; further investigation in larger cohorts is warranted.

## 1 Introduction

Encephalopathy, lethal, due to defective mitochondrial and peroxisomal fission 1 (EMPF1) is a rare mitochondrial disorder first described by Waterham et al. in 2007 (1). The condition is caused by mutations in the DNM1Lgene, which encodes dynamin-related protein 1 and plays a critical role in mediating mitochondrial fission. Pathogenic variants disrupt fission kinetics and impair organelle function, thereby contributing to the disease pathogenesis (2–4). Clinically, EMPF1 is characterized by acute-onset refractory seizures that rapidly evolve to status epilepticus, accompanied by precipitous cognitive and motor regression, culminating in a poor prognosis (5, 6). Conventional treatments, including antiepileptic drugs and the ketogenic diet, have shown limited efficacy (7). Although non-invasive neuromodulation approaches such as repetitive transcranial magnetic stimulation (rTMS) have demonstrated benefit in other drug-resistant epilepsies, their application in EMPF1 remains unexplored. We herein describe a pediatric patient with DNM1L-associated EMPF1 who exhibited a temporal association between adjunctive rTMS and a marked reduction in seizure frequency. Given the multifaceted nature of the intervention, the observed clinical response should be regarded as a hypothesis-generating signal rather than conclusive proof of efficacy.

## 2 Case presentation

A 7-year-old boy, born at term by uncomplicated vaginal delivery, had achieved all developmental milestones on schedule and was attending first grade with average academic performance. He had no prior history of epilepsy, neurodevelopmental disorders, or systemic illness, and there was no family history of epilepsy, mitochondrial disease, or unexplained early death. On admission (designated as Day 1, the first day of symptom onset), he was febrile (peak 39 °C), irritable, and nonverbal, with intermittent right-hand clonic movements. Initially, he presented with fever, abdominal pain, and recurrent vomiting following a meal. The following day, he developed right-hand clonic movements accompanied by impaired consciousness. Although a convulsive episode was terminated by diazepam injection, the patient remained irritable and nonverbal. Laboratory investigations revealed leukocytosis with neutrophilic predominance; coagulation profiles, routine biochemistry, and toxicology screening were unremarkable. Cranial CT showed no evidence of acute hemorrhage. The patient subsequently progressed to status epilepticus with declining oxygen saturation, requiring endotracheal intubation. Seizures persisted despite sedation with midazolam, propofol, and valproate, manifesting as frequent bilateral tonic-clonic convulsions accompanied by excessive oropharyngeal secretions and fever. He was subsequently transferred to our institution for further management.

Following admission, laboratory studies revealed elevated inflammatory markers (C-reactive protein and procalcitonin). Empirical antimicrobial therapy with cephalosporins and acyclovir was initiated, alongside mannitol for intracranial pressure management. Brain MRI demonstrated diffuse cytotoxic edema involving the bilateral frontal, parietal, and temporal cortices, basal ganglia, and thalami (Figure 1a). EEG exhibited generalized periodic spike-wave discharges with marked background slowing (Figure 1b). Seizure control was attempted with continuous intravenous infusions of midazolam (10 μg/kg/min), propofol (1 mg/kg/h), phenobarbital (2.08 mg/kg/day), and valproate (1 mg/kg/h). Cerebrospinal fluid analysis showed normal cell counts, protein levels, and immunoglobulin profiles. Autoantibody panels for encephalitis and demyelinating disorders (EUROIMMUN, FA112d-1, Germany) were negative, and cerebrospinal fluid (CSF) metagenomic next-generation sequencing detected no pathogens. High-dose methylprednisolone pulse therapy (25 mg/kg/day for 5 days) and intravenous immunoglobulin (IVIG; 0.4 g/kg/day for 5 days) were administered. Although a modest reduction in tonic-clonic seizure frequency was observed, new-onset myoclonic seizures—predominantly affecting the head and right limbs—emerged, with approximately 20 episodes per day. Despite the subsequent addition of clobazam and brivaracetam combined with clonazepam, seizure control did not improve significantly. A quadriceps muscle biopsy revealed no histological features suggestive of mitochondrial myopathy. Metabolic profiling revealed abnormal amino acid ratios, including increased arginine (Arg), citrulline (Cit), ornithine (Orn), Arg/Orn, Cit/phenylalanine (Phe), methionine (Met)/leucine (Leu), and tyrosine (Tyr)/Leu, with decreased valine (Val) and glutamine (Gln)/Cit. Genetic testing identified a novel *de novo* DNM1L mutation (c.1949T > C, p.Leu650Pro, NM_012062.5), Trio whole-exome sequencing (average depth >100×) was performed on the patient and both parents. Sanger sequencing confirmed that neither parent carried the variant, establishing its *de novo* origin. The variant was interpreted using VarSome and classified as likely pathogenic according to ACMG/AMP 2015 guidelines based on the following criteria: PS2_Moderate (*de novo* in a patient with a phenotype highly specific for a DNM1L-related disorder), PM2_Supporting (absent from gnomAD v2.1.1 and v3.1.2), PM5 (missense substitution at the same codon as a previously reported pathogenic variant p.Leu650Gln), PP2 (missense variant in a gene where such variants are a common mechanism of disease), and PP3 (multiple in silico predictors, including REVEL, PolyPhen-2, and SIFT, consistently predicted a deleterious effect). These findings, together with the clinical presentation and auxiliary test results, provided strong support for the diagnosis of encephalopathy, lethal, due to defective mitochondrial and peroxisomal fission 1 (EMPF1). In terms of the differential diagnosis, the initial presentation with febrile gastrointestinal illness followed by refractory status epilepticus prompted consideration of several conditions. Infectious encephalitis was unlikely given normal CSF parameters, negative mNGS, and lack of response to empirical antimicrobials. Autoimmune encephalitis was considered but deemed improbable due to negative CSF autoimmune panels and minimal response to immunotherapy. FIRES/NORSE-spectrum presentations were entertained, but the identification of a pathogenic DNM1Lvariant provided a specific genetic etiology. Other mitochondrial disorders (e.g., POLG-related) were excluded by genetic testing. Metabolic encephalopathy was suggested by abnormal amino acid ratios, but muscle biopsy did not reveal classical mitochondrial myopathy, and the genetic diagnosis superseded.

**Figure 1:**
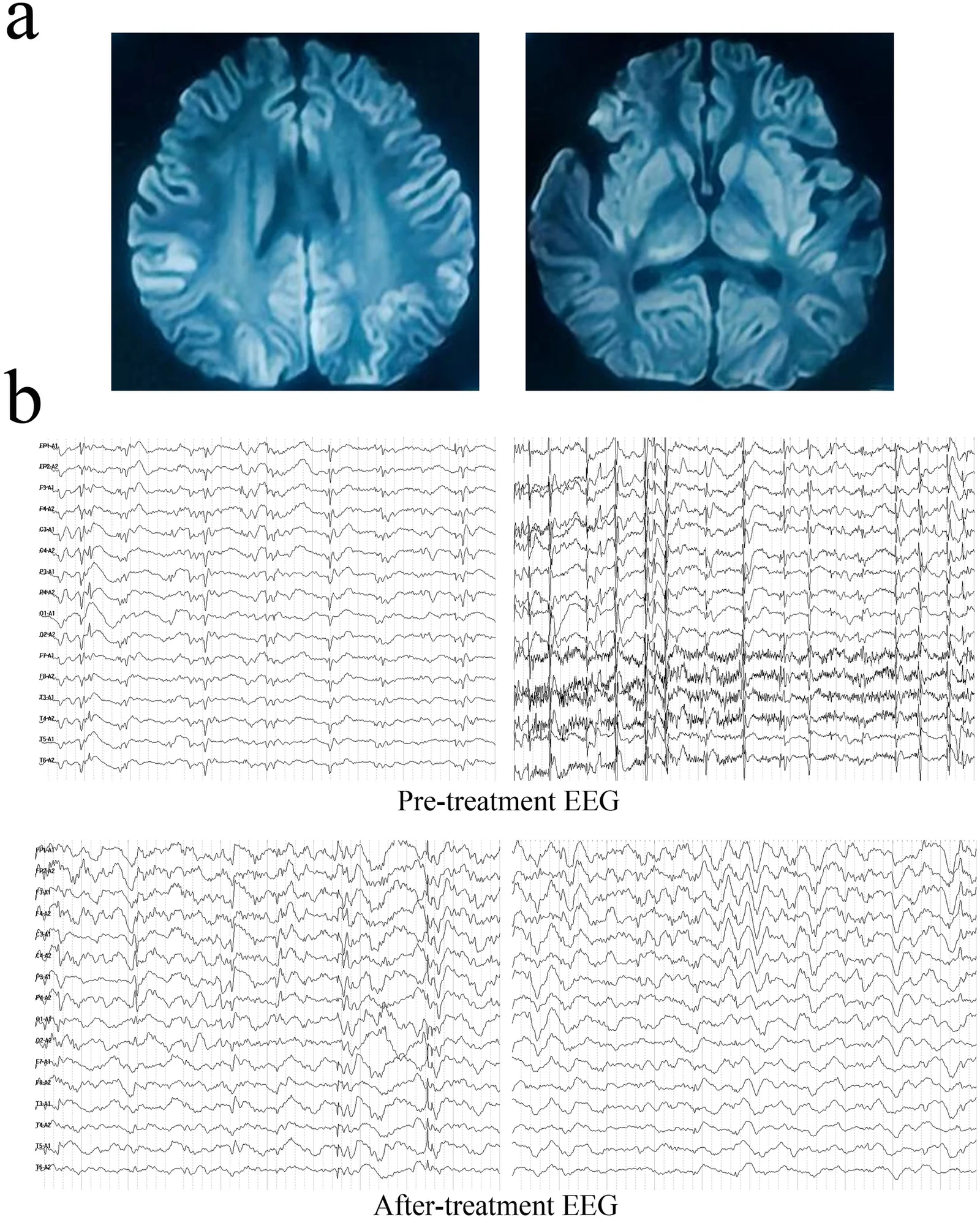
**(a)** brain MRI features of the patient. Axial diffusion-weighted imaging (DWI, b = 1000 s/mm^2^) shows diffuse high signal involving bilateral frontal, parietal, and temporal cortices, basal ganglia, and thalami. Corresponding apparent diffusion coefficient (ADC) maps (not shown) demonstrated low signal, confirming cytotoxic edema. **(b)** The EEG results of the patient before and after rTMS. Pre-rTMS EEG (recorded on Day 15) showed generalized periodic spike-wave discharges with background slowing. Post-rTMS EEG (recorded on Day 36, after 21 sessions) shows a marked reduction in epileptiform discharges compared to the pre-rTMS recording. EEG technical parameters: montage, average reference; sensitivity, 100 *μ*V/cm; time scale, 1 s/div.

A metabolic “cocktail” therapy—consisting of coenzyme Q10 (100 mg three times daily), vitamin B1 (100 mg twice daily), acetylcysteine (500 mg three times daily), and idebenone (30 mg three times daily)—was initiated. Despite this, myoclonic seizures persisted. During subsequent video-EEG monitoring, the patient was also observed to experience stimulus-induced clonic seizures involving the head and both upper extremities. Given the patient's focal right-hand clonic seizures and the constraints of endotracheal intubation, bedside repetitive transcranial magnetic stimulation (rTMS) was administered targeting the left primary motor cortex (M1).

Stimulation was delivered using a figure-eight coil connected to an rTMS stimulator (Magstim Company Ltd., UK). Parameters were set as follows: frequency 1 Hz, intensity 90% of the resting motor threshold (RMT). RMT was determined by electromyographic recording from the right first dorsal interosseous muscle during minimal sedation (midazolam infusion at 2 μg/kg/min), using visual and auditory feedback. The coil was positioned over the left primary motor cortex (M1) using the international 10–20 EEG system (C3 position), with the coil handle oriented posteriorly; neuronavigation was not used. The decision to target left M1 was based on the clinical semiology of right-hand clonic onset and neuroimaging findings suggestive of left hemispheric involvement, although EEG-defined seizure-onset localization was not available to confirm this clinical localization. Each daily session consisted of 40 trials, with each trial comprising a 30-pulse train; trials were separated by a 1-second interval. The total daily stimulation duration was 20 min. No rTMS-related adverse events were observed, including no stimulation-associated seizure worsening, autonomic instability, or scalp discomfort. It should be noted that the decision to apply rTMS over the left M1 in a patient with diffuse cytotoxic edema and generalized periodic discharges carries a theoretical safety concern, as the targeted cortex was acutely injured. However, no stimulation-associated adverse events were observed. The rationale for left M1 targeting was based on clinical semiology (right-hand clonic onset) and neuroimaging findings suggestive of left hemispheric involvement, rather than EEG-defined seizure-onset localization. Given the diffuse nature of the cortical injury, the applicability of standard inhibitory rTMS protocols developed for focal epilepsy may be limited in this setting. Written informed consent for off-label use of rTMS was obtained from the legal guardians, approved by the hospital ethics committee. Following the initiation of adjunctive rTMS (started on Day 16), a marked reduction in seizure frequency was observed. After a single day of rTMS treatment, myoclonic seizures decreased from approximately 20 to 5–6 episodes per day. By Day 21, phenobarbital and propofol infusions were successfully tapered while background antiepileptic medications were maintained. Seizure frequency further declined to 2–3 episodes per day by Day 27 (12 days after rTMS initiation). As the patient's level of consciousness improved, sedative agents were discontinued, and the rTMS course was concluded on Day 36 (21 days after initiation), at which time he had received a total of 21 sessions and experienced only 0–1 myoclonic event daily. Follow-up electroencephalography (EEG) on Day 36 performed during this period showed a marked reduction in epileptiform discharges compared to the initial recording (Day 15) (Figure 1b). Seizure frequency was assessed through a combination of video-EEG monitoring, nursing charts, and clinician observation, providing a more objective measure than caregiver diaries alone; however, in an intubated, sedated, encephalopathic child with mixed seizure types (including stimulus-induced seizures), clinical seizure counts remain inherently subject to underestimation of subclinical or electrographic seizures and potential double-counting. The EEG comparisons (pre-rTMS and post-rTMS) were based on single-timepoint routine EEGs rather than continuous video-EEG quantification of ictal burden, which would have represented a more rigorous gold standard. A subsequent routine EEG on Day 50 of hospitalization did not demonstrate a rebound increase in epileptiform activity, with the overall burden of interictal discharges remaining stable at a low level. Clinical timeline of the patient's course was shown in Table 1. Despite achieving substantial seizure control, the patient subsequently developed progressive pneumonia, leading to sepsis and acute heart failure, and died on Day 65.

**Table 1:** Clinical Timeline of the Patient's Course.

Relative Day^a^	Clinical Events	Diagnostic Investigations	Treatments	Seizure Frequency (per day)
Day 1	Fever, abdominal pain, vomiting	CBC: leukocytosis	Supportive care	None
Day 2	Right-hand clonic movements; progressed to status epilepticus	Cranial CT: no hemorrhage	Diazepam; transferred to ICU; intubated	Frequent bilateral tonic-clonic
Day 3	Persistent status epilepticus	LP: normal CSF	Midazolam, propofol, valproate, phenobarbital; antibiotics, acyclovir, mannitol	Frequent bilateral tonic-clonic
Day 4	New myoclonic seizures (head, right limbs)	CSF mNGS: negative; autoimmune panel: negative	Methylprednisolone pulse (25 mg/kg/day) started; IVIG (0.4 g/kg/day) started	∼20 myoclonic
Day 8	Seizure frequency modestly reduced	Muscle biopsy: no mitochondrial myopathy	Clobazam, brivaracetam, clonazepam added	∼15–20 myoclonic
Day 10	Genetic result returned	WES: *DNM1L*c.1949T > C	Metabolic cocktail started	∼20 myoclonic
Day 15	Pre-rTMS EEG	EEG: generalized periodic discharges	–	
Day 16	rTMS initiated	–	rTMS (1 Hz, 90% RMT, left M1) started	∼20 myoclonic
Day 17	Seizure frequency decreased	–	rTMS continued; no change in AEDs	5–6 myoclonic
Day 21	Sedative weaning started	–	Phenobarbital and propofol tapered	2–3 myoclonic
Day 27	Further improvement; Consciousness improved	–	Remaining sedatives continued to be weaned	0–1 myoclonic
Day 36	​rTMS course concluded; post-rTMS EEG	EEG: marked reduction in epileptiform discharges	rTMS concluded (total 21 sessions); sedatives discontinued	0–1 myoclonic
Day 60	Progressive pneumonia; sepsis	Chest imaging, cultures	Antibiotics, vasopressors	0–1 myoclonic
Day 65	Death	–	Comfort care	–

a Relative days are counted from symptom onset (Day 1 = first day of fever/abdominal pain). rTMS was delivered once daily (including weekends) for 21 consecutive days (Days 16–36), totaling 21 sessions.

## 3 Discussion

Rapidly progressive, drug-resistant status epilepticus is a hallmark of this disorder and is frequently preceded by infectious illness (7, 8). In the present case, seizure onset followed a gastrointestinal infection characterized by fever, vomiting, and abdominal pain. It is hypothesized that infection-induced metabolic stress unmasks latent mitochondrial dysfunction, thereby triggering epileptogenesis. Subsequently, persistent seizures likely perpetuate a vicious cycle of energy depletion and reactive oxygen species (ROS) accumulation, further exacerbating neuronal injury.

rTMS has been widely applied in various neuropsychiatric disorders, including epilepsy, depression, and insomnia (9, 10). However, evidence for rTMS in epilepsy is derived mainly from small studies in adults with drug-resistant focal epilepsy; data in children are scarce, and reports in status epilepticus or highly refractory epilepsy are virtually absent. By modulating hyperexcitable cortical networks, rTMS is thought to suppress pathological neuronal firing and restore excitatory–inhibitory balance, thereby reducing seizure susceptibility (11). Experimental studies in stroke models further suggest that rTMS may preserve mitochondrial integrity, enhance ATP production, and attenuate oxidative stress (12, 13). However, extrapolating these findings to a primary mitochondrial fission disorder such as EMPF1 is highly speculative and should be explicitly flagged as such; the present case does not provide mechanistic evidence for such effects. The temporal association observed in this case suggests a hypothesis-generating signal for targeted cortical neuromodulation as an adjunctive therapeutic strategy in EMPF1, but the complex and evolving treatment regimen precludes attributing the observed seizure reduction primarily to rTMS.

Previous reports indicated that sedative agents such as midazolam or phenobarbital may, in some cases, transform generalized status epilepticus into focal myoclonic seizures (4, 5). Consistent with this observation, our patient developed asymmetric myoclonic status epilepticus despite ongoing sedation. Guided by the focal seizure semiology (right-hand clonic onset) and neuroimaging findings suggestive of left hemispheric involvement, rTMS was targeted to the left primary motor cortex. The subsequent reduction in seizure frequency was temporally associated with adjunctive rTMS; however, given the concurrent modifications in sedative and antiseizure regimens, this temporal association should not be interpreted as evidence that rTMS was the primary active ingredient. Future studies with larger patient cohorts and more controlled conditions are warranted to systematically evaluate the potential role of rTMS in DNM1L-related EMPF1 encephalopathy.

Several important limitations of this study must be acknowledged. First, the single-case design inherently restricts the generalizability of the findings and precludes definitive conclusions regarding therapeutic efficacy. Second, seizure frequency was assessed through a combination of video-EEG monitoring, nursing charts, and clinician observation, which provides a more objective measure than caregiver diaries alone, but still may be subject to bias. Most critically, the observed reduction in seizure frequency occurred alongside concurrent stable regimens of antiepileptic drugs and metabolic therapy. At the time of rTMS initiation, the following medications were unchanged: clobazam, brivaracetam, clonazepam, midazolam, propofol, phenobarbital, and valproate; the metabolic cocktail had been initiated 6 days prior and was stable. Phenobarbital and propofol were tapered by day 5 after rTMS initiation, and other sedatives were discontinued as consciousness improved (see Table 1 for precise timing). Therefore, the clinical improvement cannot be conclusively attributed to rTMS and may reflect combined or synergistic effects of all interventions, or even the natural fluctuation of the disease. These limitations underscore that the present findings demonstrate a temporal association rather than a proven causal effect of rTMS.

Due to the patient's critical condition and subsequent death, a formal patient/family perspective could not be obtained. However, the family provided informed consent for publication and expressed hope that sharing this experience would benefit other families facing similar challenges.

## 4 Conclusions

This case describes a temporal association between adjunctive rTMS, combined with conventional antiepileptic drugs and metabolic “cocktail” therapy, and a marked reduction in seizure frequency in a pediatric patient with DNM1L-associated EMPF1. While the single-case nature and concomitant treatments preclude definitive conclusions regarding efficacy, the observed response suggests that this multimodal approach, including targeted neuromodulation, may represent a hypothesis-generating signal for mitigating seizures in this severe disorder and warrants further investigation. Despite the reduction in clinical seizure burden, the patient ultimately died from sepsis and multisystem failure, underscoring the poor prognosis of this devastating disorder.

## Data Availability

The original contributions presented in this study are included in the article. Due to the nature of this being a single case report, no large-scale datasets were generated. Further inquiries can be directed to the corresponding authors.
